# Candidate Gene Sequencing of *SLC11A2* and *TMPRSS6* in a Family with Severe Anaemia: Common SNPs, Rare Haplotypes, No Causative Mutation

**DOI:** 10.1371/journal.pone.0035015

**Published:** 2012-04-11

**Authors:** Anita Kloss-Brandstätter, Gertraud Erhart, Claudia Lamina, Bernhard Meister, Margot Haun, Stefan Coassin, Markus Seifert, Andreas Klein-Franke, Bernhard Paulweber, Lyudmyla Kedenko, Barbara Kollerits, Dorine W. Swinkels, Sita H. Vermeulen, Tessel E. Galesloot, Florian Kronenberg, Günter Weiss

**Affiliations:** 1 Division of Genetic Epidemiology, Department of Medical Genetics, Molecular and Clinical Pharmacology, Innsbruck Medical University, Innsbruck, Austria; 2 Department of Paediatrics II, Innsbruck Medical University, Innsbruck, Austria; 3 Department of Internal Medicine I, Clinical Immunology and Infectious Diseases, Innsbruck Medical University, Innsbruck, Austria; 4 First Department of Internal Medicine, Paracelsus Medical University, Salzburg, Austria; 5 Department of Laboratory Medicine, Laboratory of Genetic, Endocrine and Metabolic Diseases, Radboud University Nijmegen Medical Centre, Nijmegen, The Netherlands; 6 Department of Epidemiology, Biostatistics and HTA, Radboud University Nijmegen Medical Centre, Nijmegen, The Netherlands; Johns Hopkins School of Public Health, United States of America

## Abstract

**Background:**

Iron-refractory iron deficiency anaemia (IRIDA) is a rare disorder which was linked to mutations in two genes (*SLC11A2* and *TMPRSS6*). Common polymorphisms within these genes were associated with serum iron levels. We identified a family of Serbian origin with asymptomatic non-consanguineous parents with three of four children presenting with IRIDA not responding to oral but to intravenous iron supplementation. After excluding all known causes responsible for iron deficiency anaemia we searched for mutations in *SLC11A2* and *TMPRSS6* that could explain the severe anaemia in these children.

**Methodology/Results:**

We sequenced the exons and exon–intron boundaries of *SLC11A2* and *TMPRSS6* in all six family members. Thereby, we found seven known and fairly common SNPs, but no new mutation. We then genotyped these seven SNPs in the population-based SAPHIR study (n = 1,726) and performed genetic association analysis on iron and ferritin levels. Only two SNPs, which were top-hits from recent GWAS on iron and ferritin, exhibited an effect on iron and ferritin levels in SAPHIR. Six SAPHIR participants carrying the same *TMPRSS6* genotypes and haplotype-pairs as one anaemic son showed lower ferritin and iron levels than the average. One individual exhibiting the joint *SLC11A2/TMPRSS6* profile of the anaemic son had iron and ferritin levels lying below the 5^th^ percentile of the population's iron and ferritin level distribution. We then checked the genotype constellations in the Nijmegen Biomedical Study (n = 1,832), but the profile of the anaemic son did not occur in this population.

**Conclusions:**

We cannot exclude a gene-gene interaction between *SLC11A2* and *TMPRSS6*, but we can also not confirm it. As in this case candidate gene sequencing did not reveal causative rare mutations, the samples will be subjected to whole exome sequencing.

## Introduction

Iron deficiency is a global health concern and usually attributed to chronic blood loss or inadequate dietary iron intake [1]. In rare cases iron deficiency can be referred to insufficient duodenal iron absorption which cannot be sufficiently treated by oral iron supplementation. This type of anaemia is termed iron-refractory iron-deficiency anaemia (IRIDA) and is an autosomal-recessive disorder, characterized by (i) congenital, hypochromic, microcytic anaemia; (ii) very low mean corpuscular volume (MCV); (iii) low serum iron and low transferrin saturation; (iv) normal ferritin or ferritin levels in the lower limits of normal; (v) no response to oral iron treatment; and (vi) inappropriately high levels of hepcidin [2], [3].

At the luminal side of the gut dietary iron is transferred into the enterocyte by the divalent metal transporter 1 (*DMT1*, *SLC11A2*) [4]. *SLC11A2* (solute carrier family 11 (proton-coupled divalent metal ion transporters), member 2) also transfers iron from the endosomes to the cytosol following the uptake of iron via a transferrin receptor complex, is also of importance for the shuttling of iron in several tissues such as the liver or kidney and importantly for the transfer of iron taken up via transferrin receptor mediated endocytosis from the endosome into the cytoplasm [4]–[6]. Dysregulation of *SLC11A2* can lead to disturbances of iron homeostasis. For example, activation of *SLC11A2* in the brain was associated with toxic iron accumulation, autophagy and cell death in mouse models of Parkinson disease [7] whereas pharmacological modulation of *SLC11A2* activity can reverse hepatic iron overload in mouse models of hemochromatosis [4]. Importantly, a loss of function mutation was responsible for the development of severe microcytic anaemia in mk/mk mice [6]. Subsequently, several rare mutations in *SLC11A2* were identified and were linked to the development of microcytic anaemia in a total of 4 patients [8]–[11]. Interestingly, such patients present with low serum ferritin levels but normal or increased transferrin saturation along with low hepcidin concentrations [8]–[12].

*TMPRSS6* (transmembrane serine protease 6) mutations were described to cause IRIDA [13]–[16]. *TMPRSS6* encodes for matriptase-2, a type II transmembrane serine protease mainly produced by the liver. *TMPRSS6* belongs to a large group of type two serine proteases which modulate a variety of cellular processes including the selective cleavage of specific substrates which is fulfilled by a conserved catalytic motif. Type two serine proteases act as membrane bound proteases, however, soluble forms – as for *TMPRSS6* – have also been described. Accordingly, *TMPRSS6* expression is increased in early embryogenesis and mislocalization of this protease has been associated with high grade prostate cancer [17].

Recently, *TMPRSS6* has been identified as a modifier of iron homeostasis because it regulates the expression of the systemic iron regulatory hormone hepcidin [18] and inhibits hepcidin activation by cleaving membrane hemojuvelin [19]. Hepcidin controls iron absorption by binding to the only known cellular iron export protein ferroportin thereby leading to ferroportin degradation and blockage of iron egress from the enterocyte into the circulation [18], [20]. In addition, hepcidin blocks the transfer of iron from macrophages into the circulation, which is the major iron source for erythropoiesis following erythrophagocytosis and re-utilization of the metal from senescent erythrocytes [20]–[22]. Thus, under physiologic conditions high levels of hepcidin as observed with iron overload reduce iron absorption from the diet. In iron deficiency, however, low iron levels inhibit hepcidin formation and thus enables iron to be transferred from the gut to the blood [18]. Part of the iron-mediated control of hepcidin can be referred to the action of *TMPRSS6* and thus functional mutations in this gene are associated with insufficient iron absorption on the basis of increased hepcidin levels [13]–[16]. In addition, in genome-wide association studies (GWAS) common variants in *TMPRSS6* were associated with alterations of serum iron status, erythrocyte volume [23]–[25], or hemoglobin levels [26], [27].

We identified a family with asymptomatic non-consanguineous parents with three of four children presenting with severe anaemia. After excluding all known causes responsible for iron deficiency anaemia we searched for mutations in *SLC11A2* and *TMPRSS6* that could explain the severe anaemia in these children.

## Materials and Methods

### Patient characteristics and evaluation of anaemia

We describe a family of Serbian origin with asymptomatic non-consanguineous parents and three out of four children suffering from IRIDA (Table 1). This disease was diagnosed in a two year old infant (son 2) characterized by *hypochromic*, *microcytic* anaemia, very low MCV, low serum iron and low transferrin saturation and very low ferritin levels. Despite oral Fe therapy 5 mg/kg/day over four months no response of reticulocytes or hemoglobin was observed. Poor compliance or incorrect medication/dose was excluded. Usual reasons for microcytic anaemia such as chronic blood loss, gastrointestinal disease-like celiac disease, thalassemia, concurrent chronic illness or sideroblastic anaemia were excluded. Further laboratory investigations of other family members revealed that also two one year old unidentical female twins suffered from iron-refractory iron deficiency anaemia (Table 1). Because of a marginally low birth weight daughter 1 (2045 g) and daughter 2 (2550 g) (gestational age 37 weeks) received iron supplements during the whole first year of life [28]. The twin daughters received four intravenous infusions of 15 mg iron(III)-hydroxide-saccharose-complex (Venofer®), Son 2 was treated with four intravenous infusions of 50 mg each. These infusions resulted in a significant rise in hemoglobin and normalization of iron status in all three patients. Laboratory parameters at baseline and after four intravenous iron infusions on day +108 and day +152, respectively, as well as serum hepcidin levels are shown in Table 1. The 7 year old son (son 1) and their parents did not suffer from anaemia (Table 1). None of the six family members received blood transfusions or recombinant human erythropoietin at least three months before study entry. Written informed consent was obtained to take additional blood samples for determination of iron parameters and genetic analyses during a routine blood draw. The study was approved by the local ethics committee at the Medical University of Innsbruck (Approval-Nr. UN3256).

**Table 1:** Laboratory parameters of the family at the time of taking of the first blood samples, and after intravenous iron administrations in the family's children suffering from IRIDA, i.e. Son 2, and Daughters 1 and 2.

		Adult reference ranges (m/f)^a^	Father	Mother	Children ref. ranges	Son 1	Son 2	Daughter 1	Daughter 2
Age [y]			41	34		7	2	1	1
Hb [g/l]	Baseline	m:135–175/	139	141	115–155	133	84	81	89
	After Fe substitution	f: 120–160					126	105	109
MCHC [g/l]	Baseline	310–370	335	326	300–600	339	296	298	304
	After Fe substitution						344	329	328
MCV [fl]	Baseline	80–100	94	83	78–102	83	56	58	57
	After Fe substitution						73	69	61
Serum iron [µg/l]	Baseline	m:11–28	17.7	18.4	22–184	22.2	2.7	2.8	<0.9
	After Fe substitution	f:7–26					16.6	18.6	5.7
Serum ferritin [µg/l]	Baseline	m:30–400/	48	33	15–200	18	<3	<3	<3
	After Fe substitution	f:15–150					41	11	25
Serum hepcidin [ng/l]^b^	Baseline	m:0.5–11.0	<1.0	<1.0	n.a.	1.6	n.a.	<1	<1
	After Fe substitution	f:0.5–15.2^b^					n.a.	n.a.	n.a.
Transferrin saturation (%)	Baseline	16–45%	29%	32%	7–46%	26%	3%	3%	<2%
	After Fe substitution						27%	21%	7%
Transferrin [mg/dl]	Baseline	200–360	244	230	200–360	336	392	408	154
	After Fe substitution						242	325	310
Leucocytes [G/l]	Baseline	4.5–11	9.9	4.2	6–17.5	7.8	8.5	9.1	21.8
	After Fe substitution						6.3	8.5	7.1
Platelets [G/l]	Baseline	150–400	375	228	150–400	401	428	665	752
	After Fe substitution						320	439	308

Notes:

a If reference values are different for males (m) and females (f), both reference values are indicated.

b Reference values can be found at www.hepcidinanalysis.com (date of consulting the website: January 24, 2012);

n.a. not available.

### Measurement of iron status

Blood samples were drawn on a routine basis and laboratory parameters, e.g. hemoglobin, red blood cell count and serum iron parameters were determined by routine laboratory tests. Serum specimens were drawn during this routine examination and stored at −80°C until determination of serum hepcidin by a combination of weak cation exchange chromatography and time-of-flight mass spectrometry (TOF-MS), using a Microflex LT matrix-enhanced laser desorption/ionisation TOF-MS platform (Bruker Daltonics, Bremen, Germany) [29]. An internal standard (synthetic hepcidin-24; Peptide International Inc., Louisville, KT, USA) was used for quantification [29], [30].

### Sequencing of exons in *SLC11A2* and *TMPRSS6*

Genomic DNA was extracted from peripheral blood collected on EDTA on a BioRobot EZ1 advanced Workstation with the EZ1 DNA blood kit (QIAGEN, Hilden, Germany) and quantified with a NanoDrop spectrophotometer (Thermo Fisher Scientific Inc., Waltham, MA).

Seventeen exons of the *SLC11A2* gene (following the nomenclature of transcript ENST00000262051, Ensembl Release 58; www.ensembl.org) were amplified in 16 PCR reactions (Table S1) and sequenced with 32 primers (Table S2). The sequenced exons and exon-intron boundaries of *SLC11A2* summed up to a total of 8678 bp. An overview of the amplification and sequencing strategy of the exons within *SLC11A2* is given in Figure S1. A detailed description can be found in Text S1.

For setting up the amplification strategy for exons in *TMPRSS6*, we used transcript ENST00000346753, which described 16 exons; however, transcript ENST00000381792 included another exon located between Exon 16 and Exon 17 in transcript ENST00000346753. Therefore, we included this exon and renamed it “Exon 16a”. The sequenced exons and exon-intron boundaries of *TMPRSS6* summed up to a total of 7075 bp. An overview of the amplification and sequencing strategy of the exons within *TMPRSS6* is given in Figure S2. Amplification and sequencing primers are given in Tables S3 and S4.

Electrophoretic separation was carried out on an ABI3130*xl* capillary sequencer with POP-7 and a 36 cm capillary array.

### Single nucleotide polymorphism (SNP) genotyping in the SAPHIR study

For replication of the identified variants, four SNPs within *SLC11A2* (rs6580779, rs161044, rs150909, rs149411) and three SNPs within *TMPRSS6* (rs11704654, rs4820268, rs855791) were genotyped in a multiplex-format with the iPLEX® Gold assay on a MassARRAY Analyzer 4 platform (SEQUENOM, Hamburg, Germany) in the SAPHIR study. The Salzburg Atherosclerosis Prevention Program in Subjects at High Individual Risk (SAPHIR) is an observational study conducted in the years 1999–2002 involving 1,770 healthy unrelated subjects: 663 females from 39 to 67 years of age and 1,107 males from 39 to 66 years of age [31], [32]. DNA was available for 1,726 samples. Study participants were recruited by health screening programs in large companies in and around the city of Salzburg. The fraction of samples that were genotyped twice for quality assurance were 4%, the genotyping discordance rate was 0.

### Replication in the NBS study

As an additional replication, the identified variants in *TMPRSS6* and *SLC11A2* were also analyzed in 1,832 samples of the Nijmegen Biomedical Study (NBS) for which both genotype data (Illumina HumanHap370CNV-DuoBeadChip) and iron and ferritin measurements were available. Details of the Nijmegen Biomedical Study (NBS) have been described before [33]. Briefly, the NBS is a population-based survey conducted by the Department of Epidemiology, Biostatistics, and HTA and the Department of Laboratory Medicine of the Radboud University Nijmegen Medical Centre, The Netherlands. Approval to conduct the study was obtained from the Radboud University Nijmegen Medical Centre Institutional Review Board.

Genotype data for SNPs rs4820268, rs855791 and rs149411 were present on the applied genome-wide BeadChip and were extracted for the purpose of this study. Genotype data for SNPs rs11704654, rs6580779, rs161044 and rs150909 were imputed (with CEU HapMap phase II as a reference sample) and extracted and transformed to hard calls using a probability threshold of 0.9.

### Statistical analyses

Expectations between two unrelated variables were tested for equality using a Mann-Whitney-U-test. To indicate the strength and direction of a linear relationship between two random variables, Spearman's rho correlation coefficient was measured. Linear regression was used to model the relationship between the seven analyzed SNPs and iron as well as ferritin levels in the SAPHIR population; adjustments were made for age and gender by adding them as additive covariates into the regression models. Both outcome variables were log-transformed, since their distribution was skewed. Therefore, p-values from single SNP and haplotype analysis are based on the log-transformed model. For better interpretability, beta-estimates of the regression models are based on the original scale of iron and ferritin. To adjust for multiple testing, Bonferroni correction was applied to the number of effective loci calculated by the algorithm proposed by Li et al. [34]. As 5 out of 7 SNPs turned out to be effective loci, we assumed p-values<0.01 as significant.

Haplotypes within *TMPRSS6* and *SLC11A2* for the family members could be derived unambiguously, since there was one family member for each locus, who was heterozygote at no more than one locus (the father for *TMPRSS6*) or who was homozygote at all loci (son 1 for *SLC11A2*). All other haplotype combinations could be concluded from these. Haplotypes in the SAPHIR study were derived using the EM algorithm implemented in the haplo.stats package in R. Subsequently, haplotypes were allocated to individuals by taking the most probable one. By using these “best guess” haplotypes, it was possible to also look at the specific haplotype combinations, which were present in the family members. Regression models were calculated for the specific haplotype-pairs in *TMPRSS6* and *SLC11A2*, and for the combination of haplotype pairs in both genes. For the haplotype pairs in *SLC11A2* and *TMPRSS6*, the regression was performed on all haplotype pairs in one model with the most probable haplotype pair as the reference. For the combined analysis, the particular haplotype-pairs of interest were tested versus all other pairs. Each of the regression models (on iron and ferritin) was adjusted for age and sex. In the haplotype analysis, for both genes, all haplotype pairs were tested in one regression model against a reference haplotype pair. Therefore, within each haplotype regression model, there was no need for correcting for multiple testing anyway.

Statistical analyses were performed with SPSS (version 18, SPSS Inc., Chicago, Illinois) and R (version 2.14.1).

### Bioinformatic analysis

Following functional considerations based on the SNP position, the potential effects of the 7 SNPs were investigated using selected bioinformatic applications [35]. An extended description can be found in Text S2.

## Results

In the present paper, we report the clinical case of three siblings with severe iron deficiency anaemia being refractory to oral iron substitution. Given the wealth of publications describing mutations in two genes (*SLC11A2* and *TMPRSS6*) being associated with iron deficiency anaemia [8]–[10], [13], [15], [16], [36], [37], we initially hypothesized that mutations in these two candidate genes were the reason for this anaemia. Sequencing of the exons and exon–intron boundaries of both *SLC11A2* and *TMPRSS6* revealed seven known SNPs to occur in the family, but no novel mutations. No allele clearly segregated with the IRIDA trait in this family.

Four SNPs were found within *SLC11A2*: rs6580779, rs161044, rs150909, and rs149411. All family members except for the first son were heterozygotes for these SNPs (Table 2). Given the length of 8678 bp of non-redundant sequence information, we found one SNP in 2170 bp. Within the entire gene region of *SLC11A2* of ∼50 kb, ∼730 SNPs were found in dbSNP (build 135).

**Table 2:** SNP genotypes of the family within the two genes *SLC11A2* and *TMPRSS6*.

	*SLC11A2*				*TMPRSS6*		
	rs6580779	rs161044	rs150909	rs149411	rs11704654	rs4820268	rs855791
Chromosome:Base pair position	12:51420164	12:51382232	12:51381077	12:51380232	22:37499386	22:37469591	22:37462936
Located in:	5′UTR	Intron	3′UTR	3′UTR	Exon 2	Exon 13	Exon 17
Ancestral/derived allele:	A/C	C/T	T/C	A/G	C/T	A/G	G/A
SNP effect:	-	-	-	-	Syn.	Syn.	V736A
Father	1	1	1	1	0	1	2
Mother	1	1	1	1	2	1	1
Son 1	0	0	0	0	1	0	1
Son 2	1	1	1	1	1	1	2
Daughter 1	1	1	1	1	1	0	1
Daughter 2	1	1	1	1	1	1	1
Coding of genotypes							
0 =	AA	CC	TT	GG	CC	AA	GG
1 =	AC	CT	TC	GA	CT	AG	GA
2 =	CC	TT	CC	AA	TT	GG	AA

Although mutations in *TMPRSS6* are described to be linked to severe iron deficiency anaemia in patients with relatively increased hepcidin levels, we decided to sequence the exons and exon–intron boundaries of the *TMPRSS6* gene. We found three SNPs: rs11704654, rs4820268, and rs855791 (Table 2). Given the length of 7075 bp of non-redundant sequence information, we found one SNP in 2358 bp. Within the entire gene region of *TMPRSS6* of ∼44 kb, ∼784 SNPs were described in dbSNP (build 135). The SNP rs855791 results in a nonsynonymous (V736A) change in the serine protease domain of *TMPRSS6* and was recently described as the top-hit of genome-wide association studies (GWAS) to be associated with alterations of serum iron, transferrin saturation, erythrocyte mean cell volume, blood hemoglobin levels, and glycated hemoglobin [23], [26], [38]. Similarly, rs4820268, located in exon 13, was associated with lower serum iron concentrations, lower hemoglobin levels, smaller red cells, and more variability in red cell size [24]. This variant was also discussed to have a lowering effect on hepcidin levels in urine [25].

We genotyped the seven SNPs in the population-based SAPHIR study (n = 1,726) and replicated them in NBS (n = 1,832) and found that the *TMPRSS6*-SNPs were very frequent in these populations, but that the three SNPs in *SLC11A2* occurred at minor allele frequencies of 6.1% (Table 3). In addition, the SNPs rs6580779, rs161044, and rs150909 were highly correlated with each other, although they were dispersed over the entire gene region of ∼40 kb (Figure S3). Due to this correlation structure, the number of effective loci was calculated to be 5 out of the 7 SNPs. Therefore, the significance level for the single genotype analysis was set to 0.01.

**Table 3:** SNP genotyping results in SAPHIR (n = 1,726) and NBS (n = 1,832).

	*SLC11A2*				*TMPRSS6*		
Genotype	rs6580779	rs161044	rs150909	rs149411	rs11704654	rs4820268	rs855791
0	88.1/88.7%	88.0/88.7%	88.0/88.7%	34.9/38.1%	66.4/66.2%	30.2/27.1%	33.0/29.1%
1	11.6/10.7%	11.7/10.7%	11.7/10.7%	49.3/46.5%	29.8/30.1%	48.9/51.5%	48.0/50.8%
2	0.3/0.6%	0.3/0.6%	0.3/0.6%	15.7/15.4%	3.9/3.7%	20.9/21.4%	19.0/20.1%
MAF	6.1/6.0%	6.1/6.0%	6.1/6.0%	40.4/38.7%	18.8/18.7%	45.4/47.2%	43.0/45.5%
CR	99.2/99.9%	99.7/99.9%	99.7/99.9%	99.5/99.9%	99.7/95.6%	98.8/99.7%	98.7/100%
HWE	0.348/0.089	0.539/0.089	0.538/0.089	0.311/0.431	0.313/0.638	0.552/0.159	0.408/0.323

Notes:

The coding of genotypes is indicated in Table 2. SAPHIR data are indicated on the left side of the slash, and NBS data are given on the right side of the slash.

MAF… minor allele frequency.

CR… call rate (genotyping success rate).

HWE… p-value for test for Hardy-Weinberg-Equilibrium.

When performing genetic association analysis of these seven SNPs with either iron or ferritin levels in the SAPHIR population, we found that only the two top-hit SNPs of recent GWAS on iron levels [23], [24], [26] (rs4820268 and rs855791) exhibited a slight, though significant effect on iron and ferritin (Table 4). These associations remain significant even after correction for multiple testing. The remaining five SNPs showed no influence on the analyzed outcome variables. Since rs4820268 and rs855791 were highly correlated (D' = 0.95, r^2^ = 0.83), they rather reflect the same information. In order to test for a possible interaction of the only two significant SNPs rs4820268 and rs855791 on both iron and ferritin levels, we introduced the interaction term rs4820268*rs855791 to the linear regression model, but it was neither significant on iron (p = 0.469) nor on ferritin levels (p = 0.686). Therefore, we could not detect an epistatic effect of these two SNPs on iron-related traits.

**Table 4:** Genetic association between iron and ferritin levels and SNPs in *SLC11A2* and *TMPRSS6* in SAPHIR.

	Iron [µg/dl]						Ferritin [µg/l]					
SNP	AA	Aa	aa	β	95% CI	p-value	AA	Aa	aa	β	95% CI	p-value
rs6580779 (*SLC11A2*)	100.23	98.82	97.41	−1.41	(−6.11–3.29)	0.567	175.30	166.62	157.94	−8.68	(−35.03–17.67)	0.108
rs161044 (*SLC11A2*)	100.23	98.57	96.90	−1.67	(−6.34–3.01)	0.506	175.77	167.09	158.40	−8.68	(−34.86–17.50)	0.102
rs150909 (*SLC11A2*)	100.25	98.57	96.89	−1.68	(−6.36–2.99)	0.503	175.51	167.02	158.53	−8.48	(−34.66–17.69)	0.107
rs149411 (*SLC11A2*)	100.76	99.84	98.93	−0.91	(−3.19–1.37)	0.564	175.80	174.55	173.29	−1.25	(−14.07–11.56)	0.534
rs11704654 (*TMPRSS6*)	100.44	99.40	98.37	−1.03	(−3.83–1.78)	0.675	173.97	175.99	178.01	2.02	(−13.65–17.70)	0.569
rs4820268 (*TMPRSS6*)	104.61	99.58	94.54	−5.03	(−7.24–−2.82)	3.9E-6	191.17	172.98	154.78	−18.19	(−30.62–−5.75)	0.001
rs855791 (*TMPRSS6*)	104.60	99.41	94.22	−5.19	(−7.40–−2.96)	2.0E-6	187.97	171.86	155.75	−16.10	(−28.60–−3.61)	0.002

Note: For linear regression, an additive genetic effect of the SNPs on the outcome variable was assumed; linear regression analyses were adjusted for age and gender. The p-values were based on the log-transformed model, and the β-estimates were based on the model on the original scale.

The haplotypes of all family members within *TMPRSS6* and *SLC11A2* are summarized in Table S5 together with the haplotype frequencies in SAPHIR. Interestingly, the most common *TMPRSS6*-haplotypes in SAPHIR was not observed in the family members. When combining the haplotypes to haplotype pairs as observed in family members, the *TMPRSS6* haplotype-pair of son 2 showed significantly reduced iron values as well as ferritin levels (Table 5). The corresponding individuals from the SAPHIR population (n = 6) showed markedly reduced iron as well as ferritin values than the average SAPHIR population (Table S6).

**Table 5:** Genetic association between iron and ferritin levels and haplotype-pairs in *SLC11A2* and *TMPRSS6* in SAPHIR.

Gene	Family member	Haplotype pair	N	Iron		Ferritin	
				β	p-value	β	p-value
*SLC11A2*	Reference	ACAC/ACAT	776	–	–	–	–
	Son 1	ACAC/ACAC	600	−0.8251	0.614	−10.2684	0.638
	All others	ACAC/CTGT	123	−1.3861	0.577	−10.1689	0.241
*TMPRSS6*	Reference	CAC/CGT	551	–	–	–	–
	Father	CGT/CAT	13	7.4050	0.32302	−15.080	0.5242
	Mother	TGT/TAC	30	−3.2822	0.85529	20.153	0.4125
	Son 1 & Daughter 1	TAC/CAT	4	2.1344	0.92762	2.234	0.5159
	Son 2	TGT/CAT	6	−34.115	0.00278	−75.317	0.0387
	Daughter 2	CGT/TAC	0	Not present	Not present		
*SLC11A2/TMPRSS6*	Father	ACAC/CTGT; CGT/CAT	1	−8.65582	0.9257	10.7409	0.712
combined	Mother	ACAC/CTGT; TGT/TAC	0	Not present	Not present		
	Son 1	ACAC/ACAC; TAC/CAT	1	−50.7302	0.0630	−131.511	0.566
	Son 2	ACAC/CTGT; TGT/CAT	1	−60.555	0.0128	−260.230	9.57e-05
	Daughter 1	ACAC/CTGT; TAC/CAT	0	Not present	Not present		
	Daughter 2	ACAC/CTGT; CGT/TAC	0	Not present	Not present		

Notes: For the haplotype pairs in *SLC11A2* and *TMPRSS6*, the regression was performed on all haplotype pairs in one model with the most probable haplotype pair as the reference. For the combined analysis, each line reflects the results from a regression model of this particular haplotype-pair versus all other pairs on the traits (each model sex- and age-adjusted). The p-values are based on the log-transformed model; the β-estimates are based on the model on the original scale.

Finally, when we combined the *TMPRSS6* and *SLC11A2* genotypes to a joint profile, we saw that the one SAPHIR individual exhibiting the profile of the anaemic son had extremely low iron and ferritin levels lying below the 5^th^ percentile of the iron and ferritin level distribution in SAPHIR. This individual exhibited also the same combination of *TMPRSS6* and *SLC11A2* haplotype-pairs as son 2, which was also associated with lower iron and ferritin levels (Table 5). Although this analysis is only based on one person, the laboratory parameters are in line with the observations made in the family.

However, this observation could not be confirmed in the Nijmegen Biomedical Study [39]. Although the NBS population was of similar size as the SAPHIR study, the *SLC11A2/TMPRSS6*-profile of the anaemic son was not observed (Table S7).

Taken together, neither the single SNP effects nor the haplotypes could explain the observed phenomenon that both the family and unrelated individuals carrying the family's joint *TMPRSS6*/*SLC11A2*-profiles in SAPHIR showed reduced iron and ferritin levels.

### Bioinformatic analysis

Bioinformatic analysis of the 7 SNPs was inconclusive and did not provide an evident candidate SNP (Table S8).

The most promising SNP for *SLC11A2* was rs6580779, which was predicted to affect a “motif ten element” (MTE), which are core promoter elements located between 18 and 27 nucleotides downstream of the transcriptional initiator element [40]. The SNP rs150909 was predicted to affect a binding site for the micro-RNA star form miR-106a* [41].

All *TMPRSS6* SNPs affected the coding region and rs855791 produced a conservative amino acid exchange, which was concordantly predicted to be benign. Also the Grantham matrix score of 64 for a Valine to Alanine substitution pointed towards a rather benign effect [42]. Furthermore, also the synonymous *TMPRSS6* SNPs were predicted to have considerable functional consequences by affecting splicing regulation elements, mRNA stability or translation efficiency [43]. Unfortunately, the in-silico prediction of these effects is, however, challenging and the performance of current algorithms for the prediction of splicing regulation elements is still improvable [44].

## Discussion

The children reported in the present study showed all features of iron deficiency anaemia, especially the inability to respond to oral iron administration. Interestingly, neither the father nor the mother was anaemic, but both were borderline iron-deficient, which is also reflected by low or undetectable serum hepcidin levels. This observation is in line with previous reports that the degree of anaemia is more evident in infancy than during adult life [16], [45].

In accordance, the results of our comparative observation with the SAPHIR study indicated that adult individuals from the SAPHIR population with identical SNP combinations and haplotypes as seen in the children of the family under investigation in this study had low iron levels but not that severe iron deficiency as seen in the children. These discrepancies might thus possibly be attributed to the fact that iron parameters of very young children were compared with adults and that the phenotype could only be seen in case of high iron demand as it occurs in early childhood and adolescence where high amounts of the metal are needed for haemoglobin synthesis and growth. This also fits to observations made in *TMPRSS6* mutated subjects which have been shown to give a more severe phenotype in young children and which becomes milder with aging [16], [36].

The results of the haplotype analyses indicated that the combined *SLC11A2/TMPRSS6* haplotype of SON 2 was significantly associated with lowered iron and highly significantly associated with lowered ferritin values in SAPHIR. Unfortunately, we could not replicate this finding, as the *SLC11A2/TMPRSS6* haplotype of SON 2 was not present in the Nijmegen Biomedical Study. However, it would be interesting to validate this finding in further populations.

Although GWAS have been very successful at identifying risk alleles for complex genetic traits, there still remains the fact that the identified risk alleles for a particular trait, together, don't fully explain the trait heritability. The results of our study fit well into this observation. Various explanations for this missing-heritability problem have been proposed, but a recent study by Song et al. [46] supports the idea that perhaps this heritability is not missing at all, or at least not completely. Typically, the risk information from multiple alleles is combined by using a multiplicative model, thereby assuming that the individual loci behave independently. Song et al., however, calculated recurrence risks to close relatives under different models and found that the results yielded higher estimated recurrence risks than were predicted under a multiplicative model, particularly when the risk alleles were rare [46]. This indicates that gene-gene interactions could account for more heritability than is often assumed. The empirical data from our family study confirm the results of Song and colleagues [46], thus implying that gene-gene interactions might account for an important section of the missing heritability of complex diseases. In addition to gene-gene interactions one has also to consider interaction of the gene products at the protein level, which is a form of epistatic interaction, as has recently been shown for *DMT1* and hepcidin [47].

With this in mind, we hypothesized that the combined impairment of *SLC11A2* and *TMPRSS6* activities by the different polymorphisms effected both transmembrane iron-flux and hepcidin regulation which in summary would lead to the iron-deficient phenotype in situations with an increased iron demand (growth phase of children).

Although our data are suggestive for a gene-gene interaction, based on the methodology used, one cannot fully exclude another gene defect that might contribute to the dramatic anaemia in the children which might be identified by whole exome sequencing.

## Supporting Information

Figure S1 **Overview of the amplification and sequencing strategy of exons within** ***SLC11A2*** **.**
(DOC)

Figure S2 **Overview of the amplification and sequencing strategy of exons within** ***TMPRSS6*** **.**
(DOC)

Figure S3 **Linkage disequilibria between the SNPs within** ***SLC11A2*** **and** ***TMPRSS6*** **.**
(DOC)

Table S1 **Primer sequences used for amplification of exons within** ***SLC11A2*** **.**
(DOC)

Table S2 **Primer sequences used for sequencing of exons within** ***SLC11A2*** **.**
(DOC)

Table S3 **Primer sequences used for amplification of exons within** ***TMPRSS6*** **.**
(DOC)

Table S4 **Primer sequences used for sequencing of exons within** ***TMPRSS6*** **.**
(DOC)

Table S5 **Frequencies of haplotypes within** ***TMPRSS6*** **and** ***SLC11A2*** **in SAPHIR and haplotype constellations of the family members.**
(DOC)

Table S6 **Iron and ferritin values (mean ± SD) in SAPHIR, depending on the** ***SLC2A11*** **,** ***TMPRSS6*** **or joint** ***SLC11A2/TMPRSS6*** **SNP genotype.**
(DOC)

Table S7 **Iron and ferritin values in NBS (mean ± SD), depending on the** ***TMPRSS6*** **,** ***SLC2A11 or*** **joint** ***TMPRSS6/SLC11A2*** **SNP genotype.**
(DOC)

Table S8 **Bioinformatic predictions for the investigated SNPs in** ***SLC11A2*** **and** ***TMPRSS6*** **.**
(DOC)

Text S1 **Sequencing of exons in** ***SLC11A2*** **and** ***TMPRSS6*** **.**
(DOC)

Text S2 **Bioinformatic analysis.**
(DOC)
