# Supplementary figures and images for: Candidate Gene Sequencing of SLC11A2 and TMPRSS6 in a Family with Severe Anaemia: Common SNPs, Rare Haplotypes, No Causative Mutation
Source: PLoS One. 2012 Apr 11;7(4):e35015. doi: 10.1371/journal.pone.0035015 (PMC3324414; doi:10.1371/journal.pone.0035015)

**Figure S1** Overview of the amplification and sequencing strategy of exons within SLC11A2


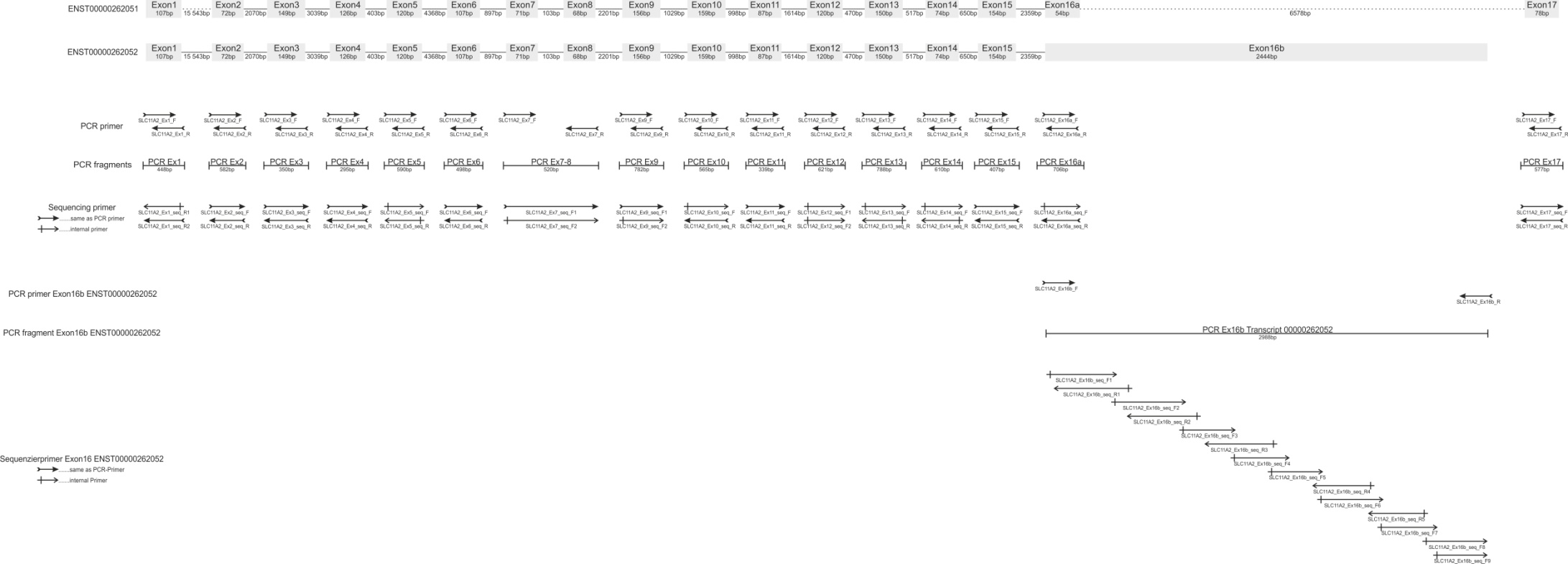

Supplement: Figure S1 — Overview of the amplification and sequencing strategy of exons within SLC11A2 . (DOC) [file pone.0035015.s001.doc]

**Figure S2** Overview of the amplification and sequencing strategy of exons within TMPRSS6


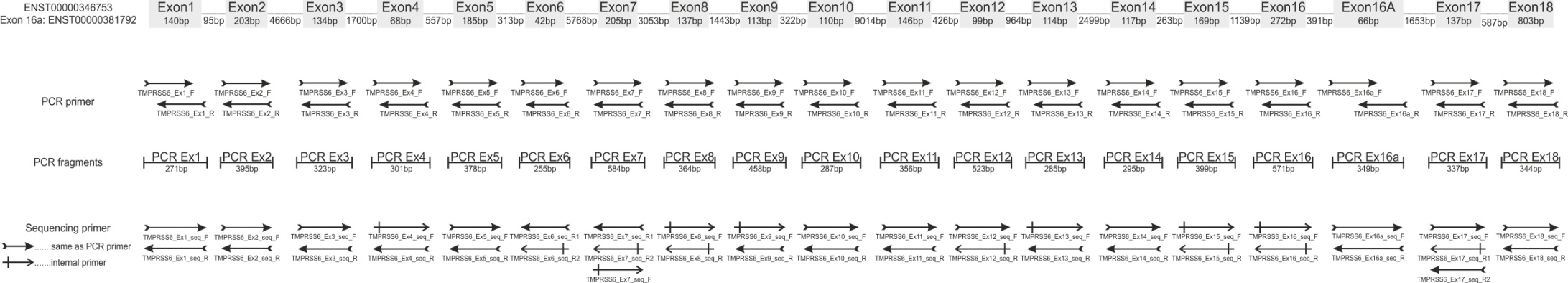

Supplement: Figure S2 — Overview of the amplification and sequencing strategy of exons within TMPRSS6 . (DOC) [file pone.0035015.s002.doc]
