# Supplementary material for: Candidate Gene Sequencing of SLC11A2 and TMPRSS6 in a Family with Severe Anaemia: Common SNPs, Rare Haplotypes, No Causative Mutation
Source: PLoS One. 2012 Apr 11;7(4):e35015. doi: 10.1371/journal.pone.0035015 (PMC3324414; doi:10.1371/journal.pone.0035015)
Supplement: Figure S3 — Linkage disequilibria between the SNPs within SLC11A2 and TMPRSS6 . (DOC) [file pone.0035015.s003.doc]

**Figure S3** Linkage disequilibria between the SNPs within *SLC11A2* and *TMPRSS6*
