# Supplementary material for: Candidate Gene Sequencing of SLC11A2 and TMPRSS6 in a Family with Severe Anaemia: Common SNPs, Rare Haplotypes, No Causative Mutation
Source: PLoS One. 2012 Apr 11;7(4):e35015. doi: 10.1371/journal.pone.0035015 (PMC3324414; doi:10.1371/journal.pone.0035015)
Supplement: Table S1 — Primer sequences used for amplification of exons within SLC11A2 . (DOC) [file pone.0035015.s004.doc]

**Table S1** Primer sequences used for amplification of exons within *SLC11A2*

| **Target** | **Primer** | **Primer sequence** | **Fragment size [bp]** |
| --- | --- | --- | --- |
| Exon 1 | SLC11A2_Ex1_F | GGTCTGGCCAACGCAAGCA | 448 bp |
|  | SLC11A2_Ex1_R | TGGCCACACCTCCCCTCACA |  |
| Exon 2 | SLC11A2_Ex2_F | TACCTCCTGTTCAATTTGTCCA | 582 bp |
|  | SLC11A2_Ex2_R | AAAAAAAAAATCCATTTTCATTT |  |
| Exon 3 | SLC11A2_Ex3_F | AGTCAGGTTGGTCTCTTGTGCATTTAGATATTA | 350 bp |
|  | SLC11A2_Ex3_R | GAACTTGAGCCATCCAGCAGATCTTT |  |
| Exon 4 | SLC11A2_Ex4_F | GGCTGGATTTTGTTGCTTCATATGTGA | 295 bp |
|  | SLC11A2_Ex4_R | ACATGCAGGGTGGAGAAAAGGATG |  |
| Exon 5 | SLC11A2_Ex5_F | GTACTTTCCATTTGGTCTTATT | 590 bp |
|  | SLC11A2_Ex5_R | TCAAGTTAGATTTTGAAAGAGGG |  |
| Exon 6 | SLC11A2_Ex6_F | GTGCAGTGATCGGCCAAGTG | 498 bp |
|  | SLC11A2_EX6_R | AAAACGCCACACATTCTAATAAAGGTAAGC |  |
| Exon 7-8 | SLC11A2_Ex7_F | GGCCTAGTTTTCTGCCATTGTC | 520 bp |
|  | SLC11A2_EX7_R | TTGGCTTTTTGTTGATACACCCACTATAA |  |
| Exon 9 | SLC11A2_Ex9_F | GTTGCAGAAGAAAAGATTTTG | 782 bp |
|  | SLC11A2_Ex9_R | TGAGGCACGAGAATCACTTGAA |  |
| Exon 10 | SLC11A2_Ex10_F | TGAAGTTCTTTTTTCTCCCC | 565 bp |
|  | SLC11A2_Ex10_R | AAACAAAAGCTGAGAAAGAGGGTCCAG |  |
| Exon 11 | SLC11A2_EX11_F | TCCCTCCAGCCTACCTCTTATAGATGATTGTG | 339 bp |
|  | SLC11A2_EX11_R | TGCTTGTCTGGTGTCTGGAACTCTGAAATAAA |  |
| Exon 12 | SLC11A2_Ex12_F | GTGTAAAGGAATATAATTTGG | 621 bp |
|  | SLC11A2_Ex12_R | TGAGTCAGGAGAATCACTTGAA |  |
| Exon 13 | SLC11A2_Ex13_F | GTTCAAGTGATTCTCCTGACTCA | 788 bp |
|  | SLC11A2_Ex13_R | GTATGTGACTTGTCTTCTTAACCT |  |
| Exon 14 | SLC11A2_Ex14_F | GCAAACATTAAATGAACATATAGGA | 610 bp |
|  | SLC11A2_Ex14_R | CAAAGAATTAAGGTGCCTCAAA |  |
| Exon 15 | SLC11A2_Ex15_F | TCTAATTCCCATCCTGTTTCCTTTTCTG | 407 bp |
|  | SLC11A2_Ex15_R | TGGTCTCTAACTCCTGGATTCAAG |  |
| Exon 16a | SLC11A2_Ex16a_F | CAGGAATTCGAGGCTTCAGTGA | 706 bp |
|  | SLC11A2_EX16a_R | TCAGCTTTTCAAAGATCCCACCCTAATC |  |
| Exon 16b | SLC11A2_Ex16b_F | AAGGAAAACCACAGACACAAAGCAGTA | 2988 bp |
|  | SLC11A2_EX16b_R | TTCCCAAGTTTCAACATGGTAATGATG |  |
| Exon 17 | SLC11A2_Ex17_F | TCCTTAGGCAGAGTGTGTCAC | 577 bp |
|  | SLC11A2_Ex17_R | GATAACTCTGGGAGTGTATGACTG |  |

Note: all exons except for Exon 16b follow the nomenclature of transcript ENST00000262051, Ensembl Release 58; Exon 16b follows the nomenclature of transcript ENST00000262052, Ensembl Release 58, [www.ensembl.org](http://www.ensembl.org/)
