# Supplementary material for: Candidate Gene Sequencing of SLC11A2 and TMPRSS6 in a Family with Severe Anaemia: Common SNPs, Rare Haplotypes, No Causative Mutation
Source: PLoS One. 2012 Apr 11;7(4):e35015. doi: 10.1371/journal.pone.0035015 (PMC3324414; doi:10.1371/journal.pone.0035015)
Supplement: Table S2 — Primer sequences used for sequencing of exons within SLC11A2 . (DOC) [file pone.0035015.s005.doc]

**Table S2** Primer sequences used for sequencing of exons within SLC11A2

| **Target** | **Primer** | **Primer sequence** |
| --- | --- | --- |
| Exon 1 | SLC11A2_Ex1_seq_R1 | GGAGCCTGACCCCCGACACA |
|  | SLC11A2_Ex1_seq_R2 | TGGCCACACCTCCCCTCACA |
| Exon 2 | SLC11A2_Ex2_seq_F | TACCTCCTGTTCAATTTGTCCA |
|  | SLC11A2_Ex2_seq_R | AAAAAAAAAATCCATTTTCATTT |
| Exon 3 | SLC11A2_Ex3_seq_F | AGTCAGGTTGGTCTCTTGTGCATTTAGATATTA |
|  | SLC11A2_Ex3_seq_R | GAACTTGAGCCATCCAGCAGATCTTT |
| Exon 4 | SLC11A2_Ex4_seq_F | GGCTGGATTTTGTTGCTTCATATGTGA |
|  | SLC11A2_Ex4_seq_R | ACATGCAGGGTGGAGAAAAGGATG |
| Exon 5 | SLC11A2_Ex5_seq_F | GCAGTATTTACCACTCATGTTGCCAAAC |
|  | SLC11A2_Ex5_seq_R | TCACTATAGAATGAGGCCAGCACATACC |
| Exon 6 | SLC11A2_Ex6_seq_F | GTGCAGTGATCGGCCAAGTG |
|  | SLC11A2_EX6_seq_R | AAAACGCCACACATTCTAATAAAGGTAAGC |
| Exon 7-8 | SLC11A2_Ex7_seq_F1 | GGCCTAGTTTTCTGCCATTGTC |
|  | SLC11A2_Ex7_seq_F2 | CTTTATCTGGAGAGCTGTTGGGAAGAGAG |
| Exon 9 | SLC11A2_Ex9_seq_F1 | CATTGTAAGGGATAAAGGATGCTAACTGTTC |
|  | SLC11A2_Ex9_seq_F2 | GTTGCAGAAGAAAAGATTTTG |
| Exon 10 | SLC11A2_Ex10_seq_F | TGGATCTCTCACTTTTCGTCTGGTCAA |
|  | SLC11A2_Ex10_seq_R | AAACAAAAGCTGAGAAAGAGGGTCCAG |
| Exon 11 | SLC11A2_Ex11_seq_F | TCCCTCCAGCCTACCTCTTATAGATGATTGTG |
|  | SLC11A2_Ex11_seq_R | TGCTTGTCTGGTGTCTGGAACTCTGAAATAAA |
| Exon 12 | SLC11A2_Ex12_seq_F1 | CAAAGAAGGGAGAAATGCCACAGG |
|  | SLC11A2_Ex12_seq_F2 | TAGGCAGAGAAACGAAGGAG |
| Exon 13 | SLC11A2_Ex13_seq_F | GCCTCTCAAAGTGCTGGGATTACAGG |
|  | SLC11A2_Ex13_seq_R | TTCATATACATTTACTGCAGACCACAACCA |
| Exon 14 | SLC11A2_Ex14_seq_F | GTGGAGGTTAAGAAGACAAGTCACATACAAAG |
|  | SLC11A2_Ex14_seq_R | GCAGCTAGCAATCACCTCTCAAAGCA |
| Exon 15 | SLC11A2_Ex15_seq_F | TCTAATTCCCATCCTGTTTCCTTTTCTG |
|  | SLC11A2_Ex15_seq_R | TGGTCTCTAACTCCTGGATTCAAG |
| Exon 16a | SLC11A2_Ex16a_seq_F | GCTGGATGTTCAAGGAAGGGAGCA |
|  | SLC11A2_Ex16a_seq_R | TCAGCTTTTCAAAGATCCCACCCTAATC |
| Exon 16b | SLC11A2_Ex16b_seq_F1 | GCTGGATGTTCAAGGAAGGGAGCA |
|  | SLC11A2_Ex16b_seq_F2 | GATTAGGGTGGGATCTTTG |
|  | SLC11A2_Ex16b_seq_F3 | CTGACAACTAACAAAACCA |
|  | SLC11A2_Ex16b_seq_F4 | GATAGAAACAGATGGTAGTA |
|  | SLC11A2_Ex16b_seq_F5 | TCAGGAGCTGTGGACTTAA |
|  | SLC11A2_Ex16b_seq_F6 | TGATTGGTGTTCGCTGTTC |
|  | SLC11A2_Ex16b_seq_F7 | GTCTCAGATTTACAAGCATT |
|  | SLC11A2_Ex16b_seq_F8 | GTTAAAAATACCCTCATACG |
|  | SLC11A2_Ex16b_seq_F9 | GCATTTTAACCAGTACTCTG |
|  | SLC11A2_Ex16b_seq_R1 | CTAGGTGTCTCTTCATTTTATA |
|  | SLC11A2_Ex16b_seq_R2 | GAGGTGGCTTTAGGAACAA |
|  | SLC11A2_Ex16b_seq_R3 | AAATCAATGACTATAAAACAG |
|  | SLC11A2_Ex16b_seq_R4 | AAGAATTTTTTTTTTGTCGTC |
|  | SLC11A2_Ex16b_seq_R5 | GCAGCACAATTATTTCATG |
| Exon 17 | SLC11A2_Ex17_seq_F | TCCTTAGGCAGAGTGTGTCAC |
|  | SLC11A2_Ex17_seq_R | GATAACTCTGGGAGTGTATGACTG |
