# Supplementary material for: Candidate Gene Sequencing of SLC11A2 and TMPRSS6 in a Family with Severe Anaemia: Common SNPs, Rare Haplotypes, No Causative Mutation
Source: PLoS One. 2012 Apr 11;7(4):e35015. doi: 10.1371/journal.pone.0035015 (PMC3324414; doi:10.1371/journal.pone.0035015)
Supplement: Table S3 — Primer sequences used for amplification of exons within TMPRSS6 . (DOC) [file pone.0035015.s006.doc]

**Table S3** Primer sequences used for amplification of exons within TMPRSS6

| **Target** | **Primer** | **Primer sequence** | **Fragment size [bp]** |
| --- | --- | --- | --- |
| Exon 1 | TMPRSS6_Ex1_F | CTGAGACCTCCGTCTGTCCTC | 271 bp |
|  | TMPRSS6_Ex1_R | TGGAAACAGCCTCGCATTTG |  |
| Exon 2 | TMPRSS6_Ex2_F | TGCCGCCTGATGTTGTTACTC | 395 bp |
|  | TMPRSS6_Ex2_R | GCCTGCTACAGTCACCCCAAG |  |
| Exon 3 | TMPRSS6_Ex3_F | GCAGGAGAAGGCATGGAAGAG | 323 bp |
|  | TMPRSS6_Ex3_R | TCCCTGTGAATGCTCCAGATG |  |
| Exon 4 | TMPRSS6_Ex4_F | AGTAGGAGCAAAGGGCACCTC | 301 bp |
|  | TMPRSS6_Ex4_R | GACATGCAGGAAGCCAAGTTC |  |
| Exon 5 | TMPRSS6_Ex5_F | CTTCTGCGTGAAGACGGACAG | 378 bp |
|  | TMPRSS6_Ex5_R | GGCCACACCACAGCTTGTTTC |  |
| Exon 6 | TMPRSS6_Ex6_F | AGACAAGGCTGGCTCCAAGG | 255 bp |
|  | TMPRSS6_Ex6_R | CCCTGCACACACAACAGAAGC |  |
| Exon 7 | TMPRSS6_Ex7_F | AGGCGTGAAGCTCAGTGTGTG | 584 bp |
|  | TMPRSS6_Ex7_R | CTAGCCGTCCTGTCTCCCAGA |  |
| Exon 8 | TMPRSS6_Ex8_F | GATGTCCAGACTCCCGTCCAC | 364 bp |
|  | TMPRSS6_Ex8_R | GAATCTTCCCTCTCCCCATCC |  |
| Exon 9 | TMPRSS6_Ex9_F | ATTTGCTGGCAGAGGTGGTAG | 458 bp |
|  | TMPRSS6_Ex9_R | GGAAACACAGAATCCCAGGTG |  |
| Exon 10 | TMPRSS6_Ex10_F | TGTTGTTAGGGAGGTGGGTTCAC | 287 bp |
|  | TMPRSS6_Ex10_R | GAGATTGGGGACTTGGGCTTC |  |
| Exon 11 | TMPRSS6_Ex11_F | AGGGAGAAATCAGGGCAGAGG | 356 bp |
|  | TMPRSS6_Ex11_R | CCTTGGTGGTTCCAGGGATG |  |
| Exon 12 | TMPRSS6_Ex12_F | GCCACAAGGGTTTGCAGGAAT | 523 bp |
|  | TMPRSS6_Ex12_R | GAGGCTGCATTGCTGGTCTGT |  |
| Exon 13 | TMPRSS6_Ex13_F | GTGATTGGTAACGTGCAATACAGC | 285 bp |
|  | TMPRSS6_Ex13_R | TGAAGCATGTAGCAGGCCTAGA |  |
| Exon 14 | TMPRSS6_Ex14_F | CTCTTCTGGCTCCATCGTTCC | 295 bp |
|  | TMPRSS6_Ex14_R | TGAGATTTCCCTCCAGCTTCC |  |
| Exon 15 | TMPRSS6_Ex15_F | TCTCCCCCTCCATCATTCTCC | 399 bp |
|  | TMPRSS6_Ex15_R | CCACCACCCTTCCCTCTATCTG |  |
| Exon 16 | TMPRSS6_Ex16_F | ACCACCAGCTAGGCGACCTTC | 571 bp |
|  | TMPRSS6_Ex16_R | GCCCAATTTGAATCCCAGCAC |  |
| Exon 16a | TMPRSS6_Ex16a_F | AGCTCCACCTGCTGTTCCTTG | 349 bp |
|  | TMPRSS6_Ex16a_R | GGGTCTGTGTCCCCAAAACTG |  |
| Exon 17 | TMPRSS6_Ex17_F | GTGGGCAGAGCAGGAGAGAAG | 337 bp |
|  | TMPRSS6_Ex17_R | GATGTGAGCAAAGGGCCAGAC |  |
| Exon 18 | TMPRSS6_Ex18_F | CCCAGTCAATTCCCAACAGTC | 344 bp |
|  | TMPRSS6_Ex18_R | GAATACTTGTCCCCCTGCTTG |  |

Note: all exons except for Exon 16a follow the nomenclature of transcript ENST00000346753; Exon 16a follows the nomenclature of transcript ENST00000381792.
