# Supplementary material for: Candidate Gene Sequencing of SLC11A2 and TMPRSS6 in a Family with Severe Anaemia: Common SNPs, Rare Haplotypes, No Causative Mutation
Source: PLoS One. 2012 Apr 11;7(4):e35015. doi: 10.1371/journal.pone.0035015 (PMC3324414; doi:10.1371/journal.pone.0035015)
Supplement: Table S4 — Primer sequences used for sequencing of exons within TMPRSS6 . (DOC) [file pone.0035015.s007.doc]

**Table S4** Primer sequences used for sequencing of exons within TMPRSS6

| **Target** | **Primer** | **Primer sequence** |
| --- | --- | --- |
| Exon 1 | TMPRSS6_Ex1_seq_F | CTGAGACCTCCGTCTGTCCTC |
|  | TMPRSS6_Ex1_seq_R | TGGAAACAGCCTCGCATTTG |
| Exon 2 | TMPRSS6_Ex2_seq_F | TGCCGCCTGATGTTGTTACTC |
|  | TMPRSS6_Ex2_seq_R | GCCTGCTACAGTCACCCCAAG |
| Exon 3 | TMPRSS6_Ex3_seq_F | GCAGGAGAAGGCATGGAAGAG |
|  | TMPRSS6_Ex3_seq_R | TCCCTGTGAATGCTCCAGATG |
| Exon 4 | TMPRSS6_Ex4_seq_F | GTAGGAAGTGGGCCTGTCTG |
|  | TMPRSS6_Ex4_seq_R | GACATGCAGGAAGCCAAGTTC |
| Exon 5 | TMPRSS6_Ex5_seq_F | CTTCTGCGTGAAGACGGACAG |
|  | TMPRSS6_Ex5_seq_R | GGCCACACCACAGCTTGTTTC |
| Exon 6 | TMPRSS6_Ex6_seq_R1 | CCCTGCACACACAACAGAAGC |
|  | TMPRSS6_Ex6_seq_R2 | ACACAACAGAAGCCATGT |
| Exon 7 | TMPRSS6_Ex7_seq_F | GTCCCCTCCTTCTGGCTC |
|  | TMPRSS6_Ex7_seq_R1 | CTAGCCGTCCTGTCTCCCAGA |
|  | TMPRSS6_Ex7_seq_R2 | TGTGTGACTTTCAACTCCCC |
| Exon 8 | TMPRSS6_Ex8_seq_F | CCACTCCCCTCCCAGAC |
|  | TMPRSS6_Ex8_seq_R | AGGGCCCTTGGATTCTACC |
| Exon 9 | TMPRSS6_Ex9_seq_F | GTGGGGTTACAAGCTGCG |
|  | TMPRSS6_Ex9_seq_R | GGAAACACAGAATCCCAGGTG |
| Exon 10 | TMPRSS6_Ex10_seq_F | TGTTGTTAGGGAGGTGGGTTCAC |
|  | TMPRSS6_Ex10_seq_R | GAGATTGGGGACTTGGGCTTC |
| Exon 11 | TMPRSS6_Ex11_seq_F | AGGGAGAAATCAGGGCAGAGG |
|  | TMPRSS6_Ex11_seq_R | CCTTGGTGGTTCCAGGGATG |
| Exon 12 | TMPRSS6_Ex12_seq_F | GCCACAAGGGTTTGCAGGAAT |
|  | TMPRSS6_Ex12_seq_R | CTCAGCTCAGAGCAGGAAAG |
| Exon 13 | TMPRSS6_Ex13_seq_F | ACACCTTCTACAGGCATCGC |
|  | TMPRSS6_Ex13_seq_R | TGAAGCATGTAGCAGGCCTAGA |
| Exon 14 | TMPRSS6_Ex14_seq_F | CTCTTCTGGCTCCATCGTTCC |
|  | TMPRSS6_Ex14_seq_R | TGAGATTTCCCTCCAGCTTCC |
| Exon 15 | TMPRSS6_Ex15_seq_F | TCTGTCTGCTTCTCCCCTTC |
|  | TMPRSS6_Ex15_seq_R | GACACAGTGCACCTCCCAC |
| Exon 16 | TMPRSS6_Ex16_seq_F | TCTGGCTATCTTGAGGGTC |
|  | TMPRSS6_Ex16_seq_R | TTCTCCAGGCCAGGTGTTAC |
| Exon 16a | TMPRSS6_Ex16a_seq_F | AGCTCCACCTGCTGTTCCTTG |
|  | TMPRSS6_Ex16a_seq_R | GGGTCTGTGTCCCCAAAACTG |
| Exon 17 | TMPRSS6_Ex17_seq_F | GTGGGCAGAGCAGGAGAGAAG |
|  | TMPRSS6_Ex17_seq_R1 | ATGTGGGCAGCATCCTTTC |
|  | TMPRSS6_Ex17_seq_R2 | GATGTGAGCAAAGGGCCAGAC |
| Exon 18 | TMPRSS6_Ex18_seq_F | CCCAGTCAATTCCCAACAGTC |
|  | TMPRSS6_Ex18_seq_R | GAATACTTGTCCCCCTGCTTG |
