# Supplementary material for: Candidate Gene Sequencing of SLC11A2 and TMPRSS6 in a Family with Severe Anaemia: Common SNPs, Rare Haplotypes, No Causative Mutation
Source: PLoS One. 2012 Apr 11;7(4):e35015. doi: 10.1371/journal.pone.0035015 (PMC3324414; doi:10.1371/journal.pone.0035015)
Supplement: Table S5 — Frequencies of haplotypes within TMPRSS6 and SLC11A2 in SAPHIR and haplotype constellations of the family members. (DOC) [file pone.0035015.s008.doc]

**Table S5** Frequencies of haplotypes within *TMPRSS6* and *SLC11A2* in SAPHIR and haplotype constellations of the family members

| **Gene** | **Haplotype** | **Frequency in SAPHIR** | **Family Member** | | | | | |
| --- | --- | --- | --- | --- | --- | --- | --- | --- |
|  |  |  |  |  |  |  |  |  |
| *TMPRSS6* | CAC | 0.45643 |  |  |  |  |  |  |
|  | CGT | 0.32433 | F |  |  |  |  | D2 |
|  | TGT | 0.09483 |  | M |  | S2 |  |  |
|  | TAC | 0.08029 |  | M | S1 |  | D1 | D2 |
|  | CGC | 0.02084 |  |  |  |  |  |  |
|  | CAT | 0.01073 | F |  | S1 | S2 | D1 |  |
|  | TGC | 0.01202 |  |  |  |  |  |  |
|  | TAT | 0.00053 |  |  |  |  |  |  |
| *SLC11A2* | ACAC | 0.59605 | F | M | S1 | S2 | D1 | D2 |
|  | ACAT | 0.34272 |  |  |  |  |  |  |
|  | CTGT | 0.06094 | F | M |  | S2 | D1 | D2 |
|  | ATGT | 0.00029 |  |  |  |  |  |  |
|  | ATGC | 0.00000 |  |  |  |  |  |  |

Notes: This table indicates the frequency of the different haplotypes in each gene in the SAPHIR population and refers also to the family members. It can be seen that the most frequent haplotype within *TMPRSS6* in SAPHIR did not occur in the family under investigation.
