# Supplementary material for: Candidate Gene Sequencing of SLC11A2 and TMPRSS6 in a Family with Severe Anaemia: Common SNPs, Rare Haplotypes, No Causative Mutation
Source: PLoS One. 2012 Apr 11;7(4):e35015. doi: 10.1371/journal.pone.0035015 (PMC3324414; doi:10.1371/journal.pone.0035015)
Supplement: Table S6 — Iron and ferritin values (mean ± SD) in SAPHIR, depending on the SLC2A11 , TMPRSS6 or joint SLC11A2/TMPRSS6 SNP genotype. (DOC) [file pone.0035015.s009.doc]

**Supplementary Table S6** Iron and ferritin values (mean ± SD) in SAPHIR, depending on the *SLC2A11, TMPRSS6* or joint *SLC11A2/TMPRSS6* SNP genotype.

| **Gene** | **Genotype** | **N** | **Iron [µg/dl]** | **Ferritin [µg/l]** |
| --- | --- | --- | --- | --- |
| *SLC11A2* | “Father”, “Mother”, “Son 2”, “Daughter 1”, “Daughter 2” | 115 | 100.5±37.6 | 185.0±196.8 |
|  | “Son 1” | 585 | 100.8±32.8 | 189.2±164.6 |
|  | **Total SAPHIR** | **1711** | **100.7±32.8** | **192.7±195.0** |
| *TMPRSS6* | “Father” | 12 | 109.1±33.0 | 226.8±102.8 |
|  | “Mother” | 30 | 96.7±30.1 | 216.2±185.5 |
|  | “Son 1” or “Daughter 1” | 4 | 102.8±40.9 | 227.3±119.1 |
|  | “Son 2” | 6 | 66.2±25.2 | 132.8±135.4 |
|  | “Daughter 2” | 223 | 101.9±31.5 | 185.8±287.2 |
|  | **Total SAPHIR** | **1689** | **100.7±32.8** | **192.4±195.6** |
| *SLC11A2/TMPRSS6*  combined | “Father” | 1 | 94.0 (<50%) | 271.0 |
|  | “Mother” | 0 | n.a. | n.a. |
|  | “Son 1” | 1 | 52.0 (<5%) | 129.0 (<50%) |
|  | “Son 2” | 1 | 42.0 (<5%) | 10.0 (<5%) |
|  | “Daughter 1” | 0 | n.a. | n.a. |
|  | “Daughter 2” | 12 | 97.8±40.3 | 124.4±68.0 |
|  | **Total SAPHIR** | **1687** | **100.8±32.8** | **192.2±195.6** |

Notes: The table indicates the absolute frequency of the family’s SNP-genotypes in *SL11A2,* *TMPRSS6* and in the combination of the two genes. Out of 1726 genotyped SAPHIR samples, 1711 showed a complete genotype for the genotyped *SLC11A2* SNPs, 1689 showed a full profile for the analyzed *TMPRSS6* SNPs, and 1687 showed a total constellation for all analyzed *SLC11A2 and TMPRSS6* SNPs. The *SLC11A2/TMPRSS6* profile of the mother and the first daughter was never observed in SAPHIR.

For *TMPRSS6*, Son 1 and Daughter 1 showed the same SNP profile; for *SLC11A2*, only Son 1 had a SNP genotype different from the rest of the family. The joint *SLC11A2/TMPRSS6* profile of the mother and the first daughter was never observed in SAPHIR. The six SAPHIR individuals carrying the *TMPRSS6* genotype of Son 2 had significantly lower iron levels than the entire population.

In brackets: indicates whether the values lie below the 5% or 50% percentile of the entire distribution in SAPHIR, respectively.
