# Supplementary material for: Candidate Gene Sequencing of SLC11A2 and TMPRSS6 in a Family with Severe Anaemia: Common SNPs, Rare Haplotypes, No Causative Mutation
Source: PLoS One. 2012 Apr 11;7(4):e35015. doi: 10.1371/journal.pone.0035015 (PMC3324414; doi:10.1371/journal.pone.0035015)
Supplement: Table S7 — Iron and ferritin values in NBS (mean ± SD), depending on the TMPRSS6 , SLC2A11 or joint TMPRSS6/SLC11A2 SNP genotype. (DOC) [file pone.0035015.s010.doc]

**Supplementary Table S7** Iron and ferritin values in NBS (mean ± SD), depending on the *TMPRSS6, SLC2A11 or* joint *TMPRSS6/SLC11A2* SNP genotype.

| **Gene** | **Haplotype** | **N** | **Iron** | **Ferritin** |
| --- | --- | --- | --- | --- |
| *SLC11A2* | “Father”, “Mother”, “Son 2”, “Daughter 1”, “Daughter 2” | 126 | 17.2±5.2 (N=126) | 170.7±161.6 (N=126) |
|  | “Son 1” | 697 | 17.5±6.1 (N=697) | 155.7±181.3 (N=690) |
|  | **Total NBS** | **1829** | **17.3±5.7 (N=1810)** | **158.4±160.8 (N=1827)** |
| *TMPRSS6* | “Father” | 21 | 14.5±4.9 (N=20) | 142.2±82.9 (N=21) |
|  | “Mother” | 31 | 15.5±4.2 (N=31) | 183.5±159.6 (N=31) |
|  | “Son 1” or “Daughter 1” | 5 | 16.0±5.8 (N=5) | 137.3±112.4 (N=5) |
|  | “Son 2” | 5 | 19.0±7.5 (N=5) | 183.3±57.1 (N=5) |
|  | “Daughter 2” | 243 | 17.1±5.4 (N=238) | 154.3±123.5 (N=242) |
|  | **Total NBS** | **1745** | **17.2±5.7 (N=1725)** | **158.6±162.6 (N=1743)** |
| *TMPRSS6/SLC11A2*  combined | “Father” | 0 | n.a. | n.a. |
|  | “Mother” | 3 | 16.0±3.5 (N=3) | 158.4±153.0 (N=3) |
|  | “Son 1” | 3 | 14.0±6.1 (N=3) | 108.8±134.9 (N=3) |
|  | “Son 2” | 0 | n.a. | n.a. |
|  | “Daughter 1” | 0 | n.a. | n.a. |
|  | “Daughter 2” | 14 | 18.4±6.3 (N=14) | 220.1±200.5 (N=14) |
|  | **Total NBS** | **1743** | **17.2±5.7 (N=1724)** | **158.7±162.7 (N=1741)** |

Notes: The table indicates the absolute frequency of the family’s SNP genotype combinations in *TMPRSS6*, *SL11A2* and in the combination of the two genes. Out of 1832 genotyped NBS samples, 1745 showed a full profile for the analyzed *TMPRSS6* SNPs, 1829 showed a complete profile for the genotyped *SLC11A2* SNPs, and 1743 showed a total constellation for all analyzed *TMPRSS6* and *SLC11A2* SNPs. For *TMPRSS6*, Son 1 and Daughter 1 showed the same SNP profile; for *SLC11A2*, only Son 1 had a genotype combination different from the rest of the family. The joint *TMPRSS6*/*SLC11A2* profile of the father, son 2 and the first daughter was never observed in NBS.

Iron levels are expressed in µmol/L; ferritin levels in µg/L.
