# Supplementary material for: Candidate Gene Sequencing of SLC11A2 and TMPRSS6 in a Family with Severe Anaemia: Common SNPs, Rare Haplotypes, No Causative Mutation
Source: PLoS One. 2012 Apr 11;7(4):e35015. doi: 10.1371/journal.pone.0035015 (PMC3324414; doi:10.1371/journal.pone.0035015)
Supplement: Table S8 — Bioinformatic predictions for the investigated SNPs in SLC11A2 and TMPRSS6 . (DOC) [file pone.0035015.s011.doc]

**Table S8** Bioinformatic predictions for the investigated SNPs in *SLC11A2* and *TMPRSS6*.

| **SNP** | **Poly-phen** | **SIFT** | **PMUT** | **Tools predicting ESRs**  **(1-5)** | **F-SNP**  **FS score / FASTSNP risk range** | **Possible effect according to F-SNP and FASTSNP** |
| --- | --- | --- | --- | --- | --- | --- |
| ***TMPRSS6*** |  |  |  |  |  |  |
| rs11704654 |  |  |  | 2 | 0.195 / 2-3 | Affection of ESRs |
| rs4820268 |  |  |  | 3 | 0.407 / 2-3 | Affection of ESRs |
| rs855791 | benign | benign | neutral | 2 | 0.396 / 2-3 | Affection of ESRs or protein secondary structure |
| ***SLC11A2*** |  |  |  |  |  |  |
| rs6580779 |  |  |  |  | 0.101 / 1-3 | Transcriptional regulation |
| rs161044 |  |  |  |  | n.a. / 0-0 |  |
| rs150909 |  |  |  |  | n.a. / 1-2 | Intronic TFBS |
| rs149411 |  |  |  |  | n.a. / 1-2 | Intronic TFBS |

Note: An empty table cell indicates that the analysis type does not apply for the SNP localization. The effects of rs150909 and rs149411 vary depending on the transcript variant (3’ UTR for ENST00000262052 and intronic for ENST00000262051).

n.a.… not available

ESR… Exonic Splicing Regulator

TFBS… Transcription Factor Binding Site

FS-Score… Functional significance score calculated by F-SNP; ranges from 0 (neutral) to 1 (functional). The cut-off value for a potentially functional SNP is 0.5 (18).

FASTSNP risk range… ranges from 0 (no effect) to 5 (very high)
