# Supplementary material for: Candidate Gene Sequencing of SLC11A2 and TMPRSS6 in a Family with Severe Anaemia: Common SNPs, Rare Haplotypes, No Causative Mutation
Source: PLoS One. 2012 Apr 11;7(4):e35015. doi: 10.1371/journal.pone.0035015 (PMC3324414; doi:10.1371/journal.pone.0035015)
Supplement: Text S1 — Sequencing of exons in SLC11A2 and TMPRSS6 . (DOC) [file pone.0035015.s012.doc]

**Supplementary Text S1: Sequencing of exons in *SLC11A2* and *TMPRSS6***

Some primers were taken from Beaumont et al. [1], others were designed in-house with Visual OMP (DNA Software, Inc., Ann Arbor, MI). Each fragment was amplified in a total reaction volume of 25 µl, containing 60 ng of DNA, 0.25 µl of AmpliTAQ Gold polymerase (Applied Biosystems, AB, Foster City, CA), 2.5 µl of PCR reaction buffer (AB), 200 µM each dNTP (Stratagene), 0.5 µM each primer, and 1.5 mM MgCl_2_. The amplification reaction was conducted on a 96-well thermal cycler (Bio-Rad Laboratories GmbH, Munich, Germany). The reaction cocktails were heated to 95°C (10 min) and then put through 35 reaction cycles: 95°C for 20 s, 60°C for 30 s, and 72°C for 1 min, followed by a final extension phase at 72°C for 10 min. In addition, exon 16b of transcript ENST00000262052, Ensembl Release 58, was amplified in a total reaction volume of 35 µl, containing 80 ng of DNA, 0.7 µl of Herculase II Fusion DNA Polymerase (Agilent Technologies, Palo Alto, CA), 7 µl of PCR reaction buffer (Agilent Technologies), 285 µM each dNTP, and 0.25 µM each primer. The reaction cocktails were heated to 95°C (2 min) and then put through 30 reaction cycles: 95°C for 20 s, 61°C for 20 s, and 72°C for 1 min 30 sec, followed by a final extension phase at 72°C for 10 min. Exon 16b was sequenced with 14 primers (**Table S2**).

The 18 exons of *TMPRSS6* were amplified in 18 PCR reactions (**Supplementary** **Table S3**) and sequenced with 40 primers (**Supplementary** **Table S4**). Primers were taken from Finberg et al. [2] and Guillem et al. [3].

Each fragment was amplified in a total reaction volume of 25 µl, containing 100 ng of DNA, 0.25 µl of Herculase II Fusion DNA Polymerase, 5 µl of PCR reaction buffer, 250 µM each dNTP, and 0.3 µM each primer. The reaction cocktails were heated to 95°C (2 min) and then put through 6 touch-down reaction cycles: 95°C for 20 s, 72°C – 3°C/cycle for 20 s, and 72°C for 30 sec, followed by 35 amplification cycles with an annealing temperature of 55°C and a final extension phase at 72°C for 10 min.

PCR primers and unincorporated dNTPs were removed by adding 2µl of ExoSAP-IT (USB, Cleveland, OH) to each 5µl of PCR product according to the manufacturer’s protocol. For cycle sequencing, 3 µl of purified PCR product were combined with the sequencing master mix (containing 1 µl BigDye Terminator v1.1 Cycle Sequencing RR mix [AB], 3 µl Sequencing Buffer [AB], 0.3 µM primer, and distilled water up to 10 µl) and cycled (after a first denaturation step of 96°C, 2 min) for 30 cycles of 30 s at 96°C, 20 s at 55°C, and 1 min at 60°C. Purification of cycle-sequencing products with Sephadex (GE Healthcare) was according to the procedure described in Brandstätter et al. [4].

Reference List

1. Beaumont C, Delaunay J, Hetet G, Grandchamp B, de MM, Tchernia G (2006) Two new human DMT1 gene mutations in a patient with microcytic anemia, low ferritinemia, and liver iron overload. Blood 107: 4168-4170.

2. Finberg KE, Heeney MM, Campagna DR, Aydinok Y, Pearson HA, Hartman KR, Mayo MM, Samuel SM, Strouse JJ, Markianos K, Andrews NC, Fleming MD (2008) Mutations in TMPRSS6 cause iron-refractory iron deficiency anemia (IRIDA). Nat Genet 40: 569-571.

3. Guillem F, Lawson S, Kannengiesser C, Westerman M, Beaumont C, Grandchamp B (2008) Two nonsense mutations in the TMPRSS6 gene in a patient with microcytic anemia and iron deficiency. Blood 112: 2089-2091.

4. Brandstätter A, Niederstätter H, Pavlic M, Grubwieser P, Parson W (2007) Generating population data for the EMPOP database - an overview of the mtDNA sequencing and data evaluation processes considering 273 Austrian control region sequences as example. Forensic Sci Int 166: 164-175.
