# Supplementary material for: Candidate Gene Sequencing of SLC11A2 and TMPRSS6 in a Family with Severe Anaemia: Common SNPs, Rare Haplotypes, No Causative Mutation
Source: PLoS One. 2012 Apr 11;7(4):e35015. doi: 10.1371/journal.pone.0035015 (PMC3324414; doi:10.1371/journal.pone.0035015)
Supplement: Text S2 — Bioinformatic analysis. (DOC) [file pone.0035015.s013.doc]

**Supplementary Text S2: Bioinformatic analysis**

The non-synonymous variant rs855791 was analyzed for effects on protein function using the Grantham matrix score [1] and several bioinformatic applications addressing protein function, post-translational modifications and protein structure (Polyphen-2 [2], SIFT [3], PMUT [4] SNPeffect [5] and HOPE [6]). The Grantham score is a substitution matrix describing the physicochemical difference between amino acids and assigning a score to each substitution. A Grantham matrix score >60 [7] corresponds to a potentially deleterious substitution, while a value >100 is considered as deleterious [8]. All coding variants were analyzed for exonic splicing regulators (ESRs) by using the integrated ranking algorithms F-SNP [9] and FASTSNP [10]. In order to overcome the difficulty of predicting splicing regulation elements these tools query different prediction algorithms and calculate a consensus risk score called “FS score” (F-SNP) respectively “risk range” (FASTSNP).

Additionally, the SNP rs6580779 was investigated regarding the affection of promoter elements using the Genomatix Software Suite (Genomatix GmbH, Munich, Germany). Finally, SNPs in the 3’ UTR were analyzed for miRNA binding sites using Patrocles [11] and hits were follow up in mirRBase [12] and microRNA.org [13].

Reference List

1. Grantham R (1974) Amino acid difference formula to help explain protein evolution. Science 185: 862-864.

2. Adzhubei IA, Schmidt S, Peshkin L, Ramensky VE, Gerasimova A, Bork P, Kondrashov AS, Sunyaev SR (2010) A method and server for predicting damaging missense mutations. Nat Methods 7: 248-249.

3. Ng PC, Henikoff S (2003) SIFT: Predicting amino acid changes that affect protein function. Nucleic Acids Res 31: 3812-3814.

4. Ferrer-Costa C, Gelpi JL, Zamakola L, Parraga I, de lC, X, Orozco M (2005) PMUT: a web-based tool for the annotation of pathological mutations on proteins. Bioinformatics 21: 3176-3178.

5. Reumers J, Conde L, Medina I, Maurer-Stroh S, Van DJ, Dopazo J, Rousseau F, Schymkowitz J (2008) Joint annotation of coding and non-coding single nucleotide polymorphisms and mutations in the SNPeffect and PupaSuite databases. Nucleic Acids Res 36: D825-D829.

6. Venselaar H, Te Beek TA, Kuipers RK, Hekkelman ML, Vriend G (2010) Protein structure analysis of mutations causing inheritable diseases. An e-Science approach with life scientist friendly interfaces. BMC Bioinformatics 11: 548.

7. Abkevich V, Zharkikh A, Deffenbaugh AM, Frank D, Chen Y, Shattuck D, Skolnick MH, Gutin A, Tavtigian SV (2004) Analysis of missense variation in human BRCA1 in the context of interspecific sequence variation. J Med Genet 41: 492-507.

8. Miller MP, Kumar S (2001) Understanding human disease mutations through the use of interspecific genetic variation. Hum Mol Genet 10: 2319-2328.

9. Lee PH, Shatkay H (2008) F-SNP: computationally predicted functional SNPs for disease association studies. Nucleic Acids Res 36: D820-D824.

10. Yuan HY, Chiou JJ, Tseng WH, Liu CH, Liu CK, Lin YJ, Wang HH, Yao A, Chen YT, Hsu CN (2006) FASTSNP: an always up-to-date and extendable service for SNP function analysis and prioritization. Nucleic Acids Res 34: W635-W641.

11. Georges M, Clop A, Marcq F, Takeda H, Pirottin D, Hiard S, Tordoir X, Caiment F, Meish F, Bibe B, Bouix J, Elsen JM, Eychenne F, Laville E, Larzul C, Milenkovic D, Tobin J, Charlier AC (2006) Polymorphic microRNA-target interactions: a novel source of phenotypic variation. Cold Spring Harb Symp Quant Biol 71: 343-350.

12. Kozomara A, Griffiths-Jones S (2011) miRBase: integrating microRNA annotation and deep-sequencing data. Nucleic Acids Res 39: D152-D157.

13. Betel D, Wilson M, Gabow A, Marks DS, Sander C (2008) The microRNA.org resource: targets and expression. Nucleic Acids Res 36: D149-D153.
